# Glioma neuron symbiosis: a hypothesis

**DOI:** 10.3389/fnins.2025.1646148

**Published:** 2025-09-30

**Authors:** Avital Schurr

**Affiliations:** Department of Anesthesiology and Perioperative Medicine, University of Louisville School of Medicine, Louisville, KY, United States

**Keywords:** cancer, energy metabolism, glioma, glucose, glycolysis, lactate, neuron, symbiosis

## Abstract

Glioma cells, just like all cancerous cells, consume substantial amounts of glucose for their energy needs, using glycolysis, an inefficient metabolic pathway (Warburg effect) to produce only two moles of adenosine triphosphate and two moles of lactate for each mole of glucose consumed. By contrast, neurons consume glucose via glycolysis and utilize its end-product lactate as the substrate of the mitochondrial tricarboxylic acid cycle and its coupled oxidative phosphorylation, a process eighteen times more efficient at adenosine triphosphate than glycolysis alone. It hypothesizes here that glioma-produced lactate is the preferred oxidative energy substrate of their surrounding neurons. Consequently, by using lactate, neurons bypass glycolysis, sparing their glucose and making it readily available for the glucose-craving cancer cells. Moreover, glioma cells’ ability to secrete glutamate, which excites glutamatergic neurons, could drive the latter to consume even more lactate, sparing more glucose. Such symbiotic exchange, especially at the initial stages of malignancy, assures the budding cancer cells an ample glucose supply ahead of the development of additional vasculature. While this hypothesis focuses on gliomas, it may also apply to other cancer types.

## Introduction

**Figure 1:**
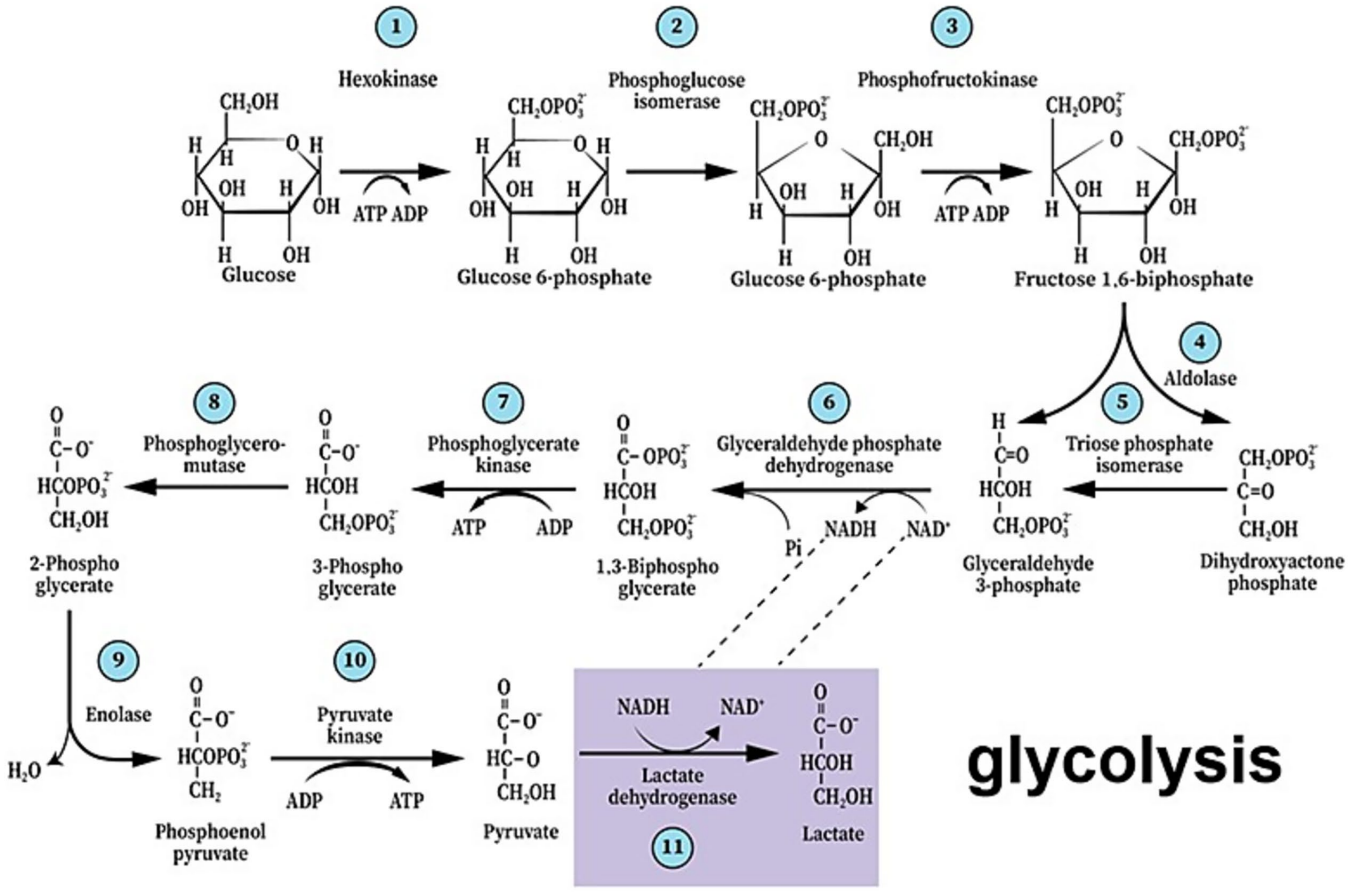
The paradigm shift of glycolysis that took place over the past four decades presents the first metabolic pathway to be elucidated as a series of 11 enzymatic reactions that begin with glucose and end with lactate, not pyruvate, independently of the presence or absence of oxygen or mitochondria. As such, it also guarantees the continuous supply of reducing power in the form of nicotinamide adenine dinucleotide (NADH), preserving the cyclical nature of the pathway.

A century ago, Otto Warburg showed that cancer cells consume substantial amounts of glucose and secrete excessive lactate (Warburg et al., 1927). This hallmark of malignancy, known as Warburg’s effect, was cited over 28,700 times since 1926 and over 5,200 times in the first 6 months of 2025, according to a Google Scholar search. The main impetus for this activity has always been the pursuit of possible anti-cancer therapy through understanding this phenomenon. Nevertheless, Warburg’s observation that cancerous cells utilize glucose glycolytically converting it to lactate, despite the presence of oxygen stood in contrast to the accepted knowledge that respirating cells converting glucose to CO_2_ and water. The general notion that lactate is a useless end-product of fermentation that also could be, at elevated levels, poisonous to normal cells, has prevailed for years. Our understanding of the relationships between cancer cells and the normal cells that surround them has grown significantly over the past half a century. Most existing research focuses on the interactions between cancerous tissue and neighboring healthy tissue in relation to tumor growth and persistence (Cuddapah et al., 2014; Venkataramani et al., 2022; Crivii et al., 2022; Gillespie and Monje, 2018). Among them, a considerable number of studies that investigated the interactions between these cell populations, deal with brain cancers, i.e., gliomas and glioblastomas. While we have a better understanding of the benefits that different brain cancer types gain from their surrounding neurons, little is known about any benefits that neurons may gain from those invading brain cancers. Where glycolysis is concerned, significant discoveries over the past four decades have ushered in a paradigm shift in this field of research (Brooks, 1985; Schurr et al., 1988; Gladden, 2004; Gladden, 2008; Hall et al., 2016). As a result, glycolysis should not be classified into two separate processes, one labeled “aerobic” with pyruvate as the end-product, and the other “anaerobic” yielding lactate as its final product, labeling that has become even more confusing these days, when aerobic glycolysis means non-oxidative glucose hydrolysis in the presence of oxygen. Rather glycolysis should be described without any prefix, just “glycolysis,” the cytosolic pathway that hydrolyzes glucose to lactate, where its last reaction, the conversion of pyruvate to lactate is catalyzed by cytosolic lactate dehydrogenase (cLDH), independently of the presence or absence of oxygen or mitochondria (Figure 1).

Lactate enters the mitochondrion via a monocarboxylate transporter (MCT), where mitochondrial lactate dehydrogenase (mLDH) transforms it back into pyruvate. The latter then enters the mitochondrial tricarboxylic acid (TCA) cycle via acetyl CoA (Passarella et al., 2008; Schurr, 2014; Rogatzki et al., 2015; Van Hall, 2000). The idea that lactate is an oxidative energy substrate for neurons has slowly gained acceptance. It is plausible that lactate is a favored mitochondrial substrate over glucose since glucose requires an investment of two moles of adenosine triphosphate (ATP) ahead of its conversion to lactate. By contrast, the oxidative utilization of lactate does not require ATP investment, and one mole of lactate produces seventeen moles of ATP through the mitochondrial TCA cycle, the electron transport chain, and its coupled oxidative phosphorylation (OXPHOS). Do neurons use glioma-secreted lactate as an oxidative mitochondrial substrate for ATP biosynthesis?

## The glioma neuron symbiosis (GNS) hypothesis: exchanging lactate for glucose

It hypothesizes that neurons regularly benefit from the endless supply of glioma-produced lactate, the preferred neuronal oxidative mitochondrial energy substrate over glucose, especially at the initial stages of malignancy, just as much as glioma cells benefit from the neuronal machinery. Moreover, the neuronal preference for lactate over glucose spares the latter, making it readily available to the glucose-craving glioma cells. Such symbiotic relationships would explain the tendency of cancerous cells (gliomas) to flourish in the vicinity of the more active brain regions (Gillespie and Monje, 2018). Contrary to the established thinking, according to which cancerous cells parasitically utilize normal cells to propagate and survive, the GNS hypothesis postulates that both glioma cells and neurons, benefit from each other, especially at the preliminary stages of metastasis, i.e., they have a symbiotic relationship.

The GNS hypothesis is based on 40 years of progress in research on brain energy metabolism. That research established lactate as the end-product of the brain glycolytic pathway, independent of the presence or absence of oxygen, demonstrating the preference of neurons, especially glutamatergic ones, to utilize it as the oxidative mitochondrial substrate. In addition, the metabolic relationship between astrocytes and neurons as laid out by the astrocyte neuron lactate shuttle (ANLS) hypothesis (Pellerin and Magistretti, 1994), and the possible origin of glioma cells from stem cells of the oligodendroglial type, both lend support to a postulated symbiotic relationship between glioma cells and neurons.

## Evolution of the hypothesis

**Figure 2:**
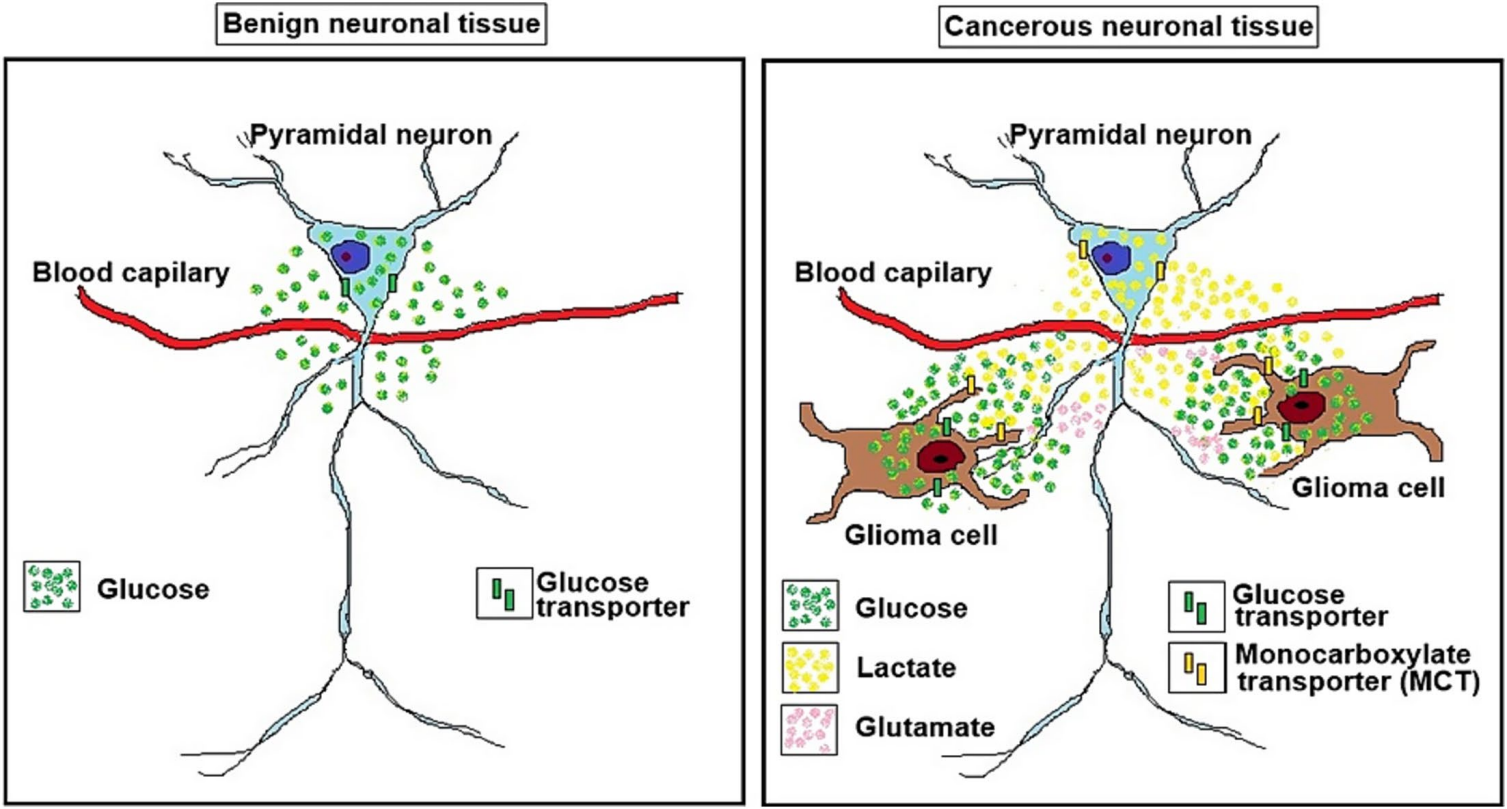
A schematic illustration of a benign neuronal tissue (left panel) and a cancerous neuronal tissue (right panel). The glioma neuron symbiosis (GNS) hypothesis as illustrated in on the right depicts the relationship between glioma cells and a glutamatergic pyramidal neuron. While in benign neuronal tissue the pyramidal neuron consumes glucose to produce ATP oxidatively via glycolysis and mitochondrial oxidative phosphorylation, the glioma cells use glycolysis to consume glucose for their ATP production, not mitochondrial oxidative phosphorylation, generating two moles of lactate for one mole of glucose consumed (the Warburg effect). The lactate is transported via monocarboxylate transporters (MCTs) from the glioma cell (MCT4 and MCT2?) to the neuron (MCT1). The neuron’s preference for lactate over glucose as its substrate for oxidative energy metabolism, which is abundantly supplied by the glioma cells, spares neuronal glucose utilization, making it available for the glioma cells. The ability of the glioma cells to secrete glutamate, the excitatory neurotransmitter that could excite the neuron, should also increase neuronal lactate consumption and would spare even more glucose for consumption by the glioma cells.

The discovery in 1988 that lactate can both support survival of brain tissue and its function *in vitro* (Schurr et al., 1988) despite the skepticism it faced for years, is now accepted universally (Gladden, 2004; Gladden, 2008; Hall et al., 2016; Passarella et al., 2008; Schurr, 2014; Rogatzki et al., 2015; Van Hall, 2000). According to the astrocyte neuron lactate shuttle (ANLS) hypothesis, published in 1994 (Pellerin and Magistretti, 1994), the excitatory neurotransmitter glutamate activates glutamatergic neurons, which thereafter is taken up by astrocytes, a function that requires the participation of the Na^+^/K^+^- ATPase pump (Pellerin and Magistretti, 1996). The ATP necessary for the pump’s action is produced by a glycolytic pathway specifically dedicated to that function. The lactate produced during this activity is transported out of astrocytes through membranal monocarboxylate transporters (MCT1 and MCT4), and into neurons via MCT2, where it is consumed oxidatively (Pellerin, 2003; Handy, 2006; Pellerin and Magistretti, 2012; Nalbandian and Takeda, 2016; Ferguson et al., 2018). Our own study demonstrated that glial cells are the source of lactate consumed by neurons (Schurr et al., 1997). Although the ANLS hypothesis is still being debated among its backers and detractors, ample evidence supports its central concept that neurons consume lactate oxidatively as the substrate of the mitochondrial TCA cycle and its coupled OXPHOS, the main source of ATP needed for neuronal function (Schurr, 2018; Schurr, 2006; Schurr, 2008; Schurr, 2023; Schurr, 2024; Bittar et al., 1996; Pellerin et al., 1998; Pellerin et al., 1998; Pierre et al., 2000; Aubert et al., 2005; Bouzier-Sore et al., 2003; Bouzier-Sore et al., 2006; Pellerin et al., 2007; Wyss et al., 2011; Proia et al., 2016; Hu and Wilson, 1997a; Schurr et al., 1999; Brooks, 2000; Brooks, 2009; Qu et al., 2000; Mangia et al., 2003; Smith et al., 2003; Kasischke et al., 2004; Herard et al., 2005; Schurr and Payne, 2007; Hashimoto et al., 2008; Erlichman et al., 2008; Gallagher et al., 2009; Chuquet et al., 2010; Figley, 2011; Dias et al., 2023). The lactate shuttle idea originated a decade before the ANLS hypothesis (Brooks, 1985) and may indicate that the phenomenon is universal. Neuronal preference for lactate over glucose has been documented both *in vitro* and *in vivo* (Hu and Wilson, 1997a; Schurr et al., 1999; Qu et al., 2000; Mangia et al., 2003; Smith et al., 2003; Kasischke et al., 2004). Taking into consideration the above-cited studies, it is reasonable to extrapolate from neuronal preference for lactate as the oxidative energy substrate to the hefty amounts of lactate glioma cells produce. Moreover, the glucose spared due to neuronal use of lactate becomes available for consumption by the glioma cells. Much understanding has been gained over the past two decades demonstrating the ability of gliomas to hijack neuronal mechanisms (Venkataramani et al., 2022; Pan and Monje, 2022; Jung et al., 2020; Tianzhen et al., 2022), flourish in the neuronal microenvironment (Crivii et al., 2022), and especially interact with active glutamatergic neurons (Gillespie and Monje, 2018). While the origin of glioma cells is still being deliberated, accumulated evidence points at “*neural stem or precursor cells of the oligodendroglial type*” (5, and references within), which could explain the ability of glioma cells to manipulate neurons for their growth needs, the very needs that are specifically being provided by active neurons (Gillespie and Monje, 2018; Buckingham et al., 2011). Clearly, in all the studies on the topic, the prevailing message is that glioma cells take over the neuronal machinery necessary for their own proliferation, a one-way relationship where these cells take all and give nothing back. However, the probable origin of glioma cells from glial cells could indicate that their interactions with neurons are like those demonstrated between astrocytes and neurons, where these two cell types have, in essence, a symbiotic relationship (Pellerin and Magistretti, 1994; Pellerin, 2003; Pellerin and Magistretti, 2012). Similarly to astrocytes, glioma cells could supply neurons with lactate. In return, the glucose spared by neurons due to their preference for lactate, is readily consumed by the glioma cells. Moreover, evidence shows that glioma cells can secrete glutamate, which excites glutamatergic neurons (Buckingham et al., 2011; Campbell et al., 2012; Venkataramani et al., 2019; Ye and Sontheime, 1999). Such excitation should increase neuronal lactate consumption and spare even more glucose for consumption by glioma cells (Figure 2). While direct communications between glioma cells and neurons have been described, including the formation of synaptic connections between the two cell types (Venkataramani et al., 2019), no reports exist on possible benefits neurons may gain through their interactions with glioma cells. Such a lack of data does not necessarily mean lack of neuronal benefits from their interaction with glioma cells. It could simply indicate that only the benefits of glioma cells were investigated, implicating a parasitic relationship between the two populations. A single review article (Turner and Adamson, 2011) alludes to the possibility that neurons interact with astrocytoma cells, where similarly to astrocytes, they extrude the lactate produced glycolytically, making it available for neuronal use. Interestingly, Sonveaux et al. (2008) demonstrated the shuttling of lactate from anaerobic cancer cells to aerobic ones. A more recent article highlights the similarities of brain tumor cells transcriptomic profiles have with oligodendrocytes and astrocytes (Pan and Monje, 2022), and a review article details the crosstalk between high grade glioma (HGGs) and their microenvironment, including neurons, astrocytes and endothelial cells (Ramachandran and Jeans, 2024). However, those interactions between tumor cells and normal brain cells are all considered to benefit the former, not the latter. Other interactions between cancer cells and neural tissue, such as glioblastoma-induced axonal injury (Hamed et al., 2025; Clements et al., 2025; Baruch et al., 2025) would be outside the scope of the present hypothesis. The absence of information on possible neuronal benefits of interaction with gliomas formed the foundation on which the GNS hypothesis has been developed. Such a symbiotic relationship, at least at the early stages of gliomas’ proliferation, benefits both cell types equally. This attraction between the two cell types is probably driven by both the glucose-hungry glioma cell and the lactate-preferred neuron. The fundamental appetite of normal and malignant cells alike for continuous supply of energy substrates could be at the basis of the interaction between other types of cancer and the normal tissues they invade. Recognizing the ability of certain cell types in other tissues and organs to efficiently utilize lactate, the glycolytic end-product of cancer cells (Warburg’s effect), could explain the tendency of certain cancer types to relocate to and proliferate in other locations. The GNS hypothesis is based on the accumulated evidence over the past four decades showing that different types of tissues and cells, when given the choice, would prefer the readily available mitochondrial substrate lactate, for their oxidative energy production over the glycolytic substrate glucose (Brooks, 1985; Schurr et al., 1988; Brooks, 2002; Brooks, 2007; Brooks et al., 2022; Brooks et al., 2022) Although where brain energy metabolism is concerned, the ANLS hypothesis appears to play a significant role in support of this preference concept, the GNS hypothesis does not stand or fall on evidence for or against the ANLS hypothesis, respectively. Moreover, the GNS hypothesis is not contingent on glutamatergic activation of neurons by glutamate secreted from glioma cells.

As mentioned earlier, Sonveaux et al. (2008) introduced the concept that anaerobic (hypoxic) cancer cells, and aerobic (normoxic) cancer cells can exist in symbiosis, with lactate produced by the anaerobic cells being oxidatively consumed by the aerobic ones. Others expanded on it, recognizing the role that lactate plays in cancer not only as a byproduct of glycolysis, but as a substrate for oxygenated cancerous cells (Semenza, 2008; Goodwin et al., 2015; Gladden, 2019; Goodwin et al., 2019; Pennington et al., 2019). The existence of two different glioma cell populations within the cancerous tumor has already brought up new suggestions for possible anti-cancer treatments (Vaupel and Multhoff, 2021; Gatto et al., 2024). Nevertheless, except for the single review article (Turner and Adamson, 2011) that alludes to the possibility that lactate produced by astrocytoma cells could be oxidatively utilized by neurons, all studies focus on either lactate production and metabolism within tumors themselves or on the hijacking of the cellular machinery of the normal cells surrounding such tumors.

## Evaluating the hypothesis and its implications

Researchers can assess the GNS hypothesis *in vitro* by monitoring lactate transport in glioma cell lines (Ye and Sontheime, 1999), either independently or in conjunction with neuronal cell lines, while manipulating MCT activity. Established MCT inhibitors, such as *α*-cyano-4-hydroxycinnamate (4-CIN) (Halestrap and Denton, 1974; Brooks et al., 1999; Schurr et al., 2001a,b) can be used to accomplish such manipulation, where the transport of lactate, extruded from cancerous cells, would be blocked and potentially prevent neuronal utilization of glioma lactate. Lactate transport blockers into neurons should also block its transport into neuronal mitochondria, consequently preventing neuronal glucose-sparing. Moreover, 4-CIN should also block lactate utilization by the normoxic glioma cells that are important for the proliferation of gliomas. An alternative *in vitro* approach would be the exposure of glioma cell lines to the glial metabolic toxin fluorocitrate (FC) (Schurr et al., 1997; Swanson and Graham, 1994). FC specifically impairs the flow of carbon through the glial cell’s TCA cycle (Swanson and Graham, 1994). Given the similarity between glial and glioma cells, the latter may react to FC in the same way as former do. When glucose availability is low, neurons co-cultured with glioma cells utilize lactate produced by the glioma cells as their primary energy source. In this case, FC may indirectly decrease neuronal survival by inducing toxic glioma cell death. Furthermore, it is possible to assess whether excitatory receptor antagonists can help reduce the impact of glutamate released by glioma cells on heightened neuronal excitability and increased lactate usage. There are multiple models for *in vivo* testing of the GNS hypothesis, each with specific strengths and limitations (Lenting et al., 2017). Presently, it would be too early to recommend one model over the other. However, in general, either a human or an animal glioma xenograft model could be employed, to evaluate the effects of MCT blockers, FC, excitatory receptor antagonists of distinct types, on both the proliferation of the glioma and on the xenograft surrounding neural tissue. Interestingly, one of the earlier studies of glioma xenografts in a mouse brain (Kaye et al., 1986) seems to be adequate for an *in vivo* evaluation of the GNS hypothesis, since it allows several pharmacological, physiological and histological manipulations. This glioma xenograft model could enable an investigation of both short- and long-term interactions between the host mouse brain tissue and the glioma xenograft. For instance, techniques that employ microsensors (Hu and Wilson, 1997b; Hu and Wilson, 1997a) to trace tissue glucose and lactate levels in the brain region (hippocampus) hosting the xenograft can be easily employed. The effects of either MCT blockers, glucose uptake transporter (GLUT) inhibitors, FC, glutamatergic antagonists or any combination thereof on the levels of the two substrates can be tested by applying these pharmacological agents locally at the microsensors via a cannula.

**Figure 3:**
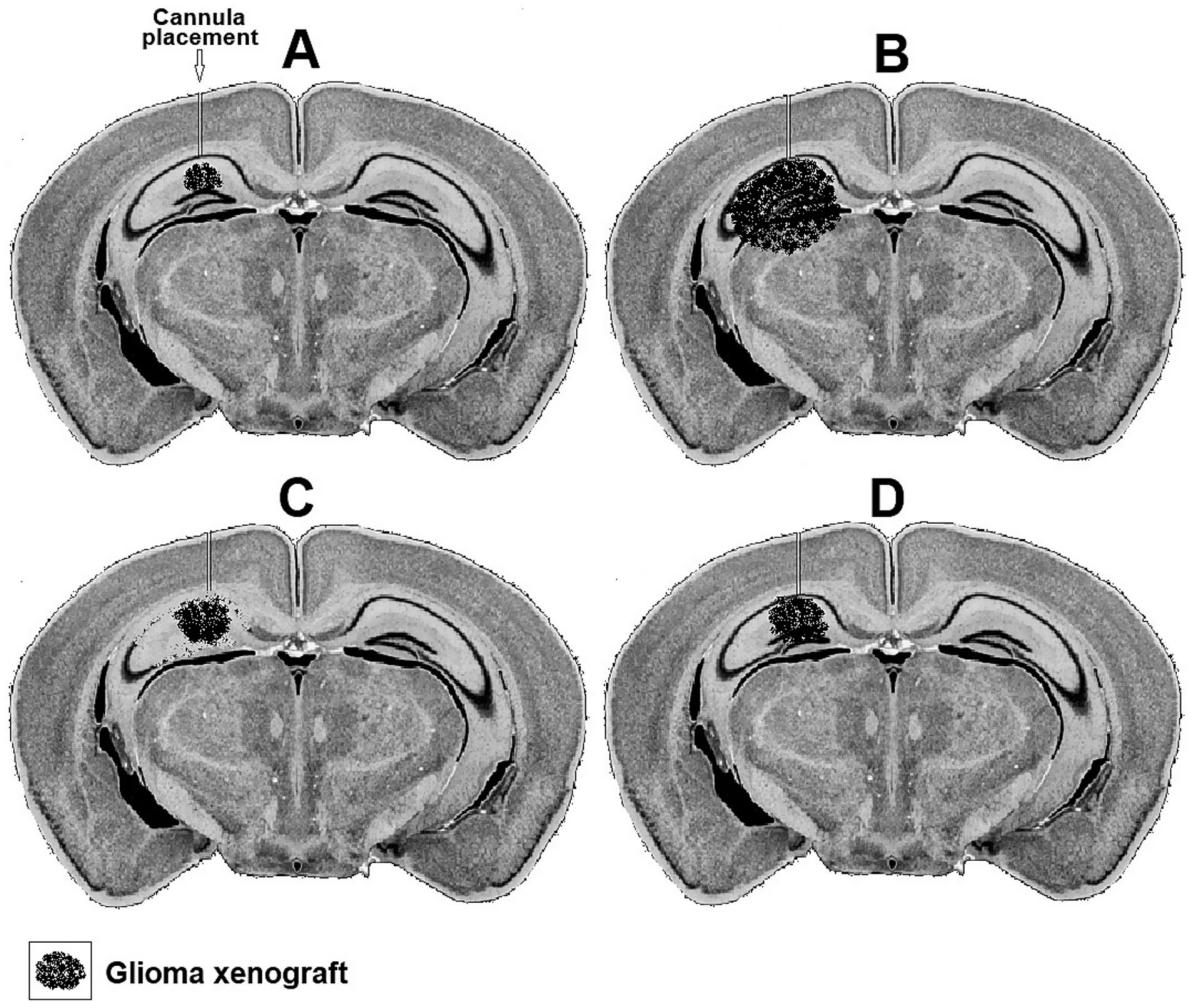
Representative hypothetical photomicrographs of brain coronal sections taken from four groups **(A–D)** of mice showing a xenograft of rat glioma a week **(A)** and 2 weeks after inoculation **(B)**. A cannula implanted close to the xenograft in the hippocampus used to deliver daily buffered solutions (vehicle) to A and B or buffered solutions containing FC **(C)** or FC + lactate **(D)** starting on day eight.

Both the short-term effects of such treatments on the levels of glucose and lactate at the interaction region of the xenograft with the hosting neuronal tissue can be measured along with their long-term effects on the rate of the xenograft proliferation. Figure 3 postulates how photomicrographs of coronal sections prepared from mice inoculated in their hippocampus with rat glioma xenograft would appear following different treatments. The progression of the glioma xenograft proliferation from 7 days (Figure 3A) to 14 days post inoculation (Figure 3B) is indicated by the enlargement of the tumor in B compared to A. Placement of a cannula in the hippocampus near the xenograft allows the delivery of chemical agents. In the example shown, the glial TCA cycle inhibitor FC was delivered daily starting on day eight after inoculation (Figure 3C) slowed down the glioma proliferation, but also damaged many pyramidal neurons in the hippocampus, since the FC is also toxic to astrocytes, the suppliers of lactate to neurons. If, however, FC was delivered along with lactate, the glioma proliferation slowed down without damaging the pyramidal neurons (Figure 3D).

The idea that glioma cells and neurons may have a symbiotic relationship indicates that there are still unexplored or unstudied areas regarding how these cells affect each other. Establishing such a symbiotic relationship between glioma cells and neurons could explain their reciprocated attraction and possible mutual dependency, at least during the early stages of proliferation. Second, if symbiosis does exist, it could open a new direction in the development of anti-cancer treatments, especially if said symbiosis is crucial for the survival and proliferation of cancerous cell types.

## Summary

The GNS hypothesis proposes that neurons regularly benefit from the endless supply of glioma-produced lactate, their preferred neuronal oxidative energy substrate over glucose. That preference spares neuronal glucose, which becomes readily available for use by the glucose-craving glioma cells. The latter’s ability to secrete glutamate, which excites glutamatergic pyramidal neurons, could increase the neuronal consumption of lactate, sparing even more glucose for consumption by glioma cells, since this excitation increases cerebral blood flow and therefore supply of glucose. Such a symbiotic relationship, at least at the beginning of the glioma’s proliferation, benefits both cell types equally. As a result, both glucose-hungry glioma cells and neurons that prefer lactate may contribute to the interaction between these two cell types. The ability of glioma cells to secrete glutamate should enhance their symbiosis with neurons. Several *in vitro* and *vivo* experiments are suggested to assess the GNS hypothesis. When the hypothesis is validated, the potential results of such validation are outlined.
